# Nutrition among nursing home residents: results from the NutriCare study

**DOI:** 10.3389/fnut.2024.1423658

**Published:** 2024-11-11

**Authors:** Živa Lavriša, Igor Pravst, Sanja Krušič, Neža Hren, Nadan Gregorič, Irena Hren, Barbara Koroušić Seljak, Hristo Hristov

**Affiliations:** ^1^Nutrition Institute, Ljubljana, Slovenia; ^2^Biotechnical Faculty, University of Ljubljana, Ljubljana, Slovenia; ^3^VIST-Faculty of Applied Sciences, Ljubljana, Slovenia; ^4^Faculty of Medicine, University of Ljubljana, Ljubljana, Slovenia; ^5^University Medical Centre Ljubljana, Ljubljana, Slovenia; ^6^General Hospital of Novo Mesto, Novo Mesto, Slovenia; ^7^Computer Systems Department, Jožef Stefan Institute, Ljubljana, Slovenia; ^8^National Institute of Public Health, Ljubljana, Slovenia

**Keywords:** older adults, nursing home residents, dietary intake, macronutrient intake, protein, energy, dietary fibre

## Abstract

**Background:**

Older adults living in nursing homes (NHs) are considered a vulnerable population in terms of nutrition. The aim of the present study was to explore the usual intake of energy, macronutrients, and specific food groups, along with offers in the NH menus on a nationally representative sample of Slovenian NH residents and compare dietary patterns with the established recommendations.

**Methods:**

The study was conducted as part of a cross-sectional NutriCare study on 317 residents (65–101 years) from 20 Slovenian NHs in 9 health regions. Relatively independent residents were selected using quota sampling by sex and age. Data collection involved interviews and anthropometric measurements. Usual dietary intake was assessed by a multiple-source method from two 24-h dietary recalls and food frequency questionnaires. Adherence of dietary intake to the recommendations and dietary composition of NH menus were assessed. The Mini Nutritional Assessment (MNA) was used to explore nutritional status.

**Results:**

Notable variability in energy and macronutrient intakes was observed with some participants showing intakes below and others above the recommended values. A high prevalence of BMI > 30 kg/m^2^ was observed in 39% of participants, indicating potential discrepancies between total energy intake and expenditure. The usual intake of fat was 36% of total energy intake (TEI). The intake of foods of animal origin exceeded dietary guidelines. Intake of carbohydrates (46% TEI in men and 47% TEI in women) as well as dietary fibre (20 g/day for both sexes) was below recommendations. A scarce intake of fruits, vegetables, and cereals was observed. In total, 40% of men and 35% of women had usual daily protein intakes lower than 1 g/kg of body weight. The protein content of breakfast and dinner could be improved. NH residents consumed little food from outside NH. The usual nutrient and food group intakes of residents reflected the NH menu offers.

**Conclusion:**

The study results on the usual intake of energy, macronutrients, specific food groups, and offers in the NH menus indicate the potential for optimisation. According to the World Health Organisation, the prevalence of BMI > 30 kg/m^2^ is notable and warrants attention. Careful meal planning and regular monitoring of the nutritional status of NH residents should be considered.

## Introduction

The global trend of an ageing population reflects a major demographic shift, characterized by an unprecedented increase in the proportion of older individuals. Addressing the needs of this population is crucial for the future overall wellbeing of society and assuring the quality of life in older years. The World Health Organisation (WHO) predicts that by 2050, the proportion of older adults, 12% of the global population in 2015, will nearly double (1). Along with these predictions, studies aiming at understanding the nutritional status of this population have gained additional focus recently.

In terms of nutrition, older adults are being recognised as a vulnerable population group, with institutionalised individuals being particularly exposed (2). An adequate intake of essential nutrients and physical activity play a major role in maintaining health of the older adults (3). Insufficient intake of nutrients increases the risk of developing health complications and loss of independence (4). The term “malnutrition” describes a lack of proper nutrition, which refers to energy, macronutrient, and micronutrient deficiencies, as well as excesses or imbalances (5). Studies report high rates of malnutrition among institutionalised older adults, which include nursing home (NH) residents (6, 7). However, the demographic structure of NH residents can vary notably. In Slovenia, for example, the structure of NH residents ranges from bed-bound, very ill, with mobility or cognitive impairment to those who are relatively independent, with low-level care requirements, who are characteristically closer to community-dwelling population or residential home inhabitants. The health characteristics of NH residents therefore have a high impact on their care requirements.

Major concerns in the nutrition of older adults are low energy and protein intake, often leading to malnutrition (8). One of the causes for the lower intake of nutrients can be also decreased appetite, which is common among older adults. In older age, one of the top priorities is maintaining muscle mass to prevent sarcopenia and frailty, which is achieved mainly through adequate protein intake and physical activity (9). The current European Society for Clinical Nutrition and Metabolism (ESPEN) guidelines for geriatrics state a daily protein intake of at least 1 g/kg body weight with up to 2 g/kg body weight/day considered appropriate based on individual health status (10). Despite this, in Europe, many institutionalised older adults have much lower daily protein intakes, which compromise muscle integrity and overall health (11). Attention should also be given to fat intake, with the recommended level set at 30% of daily energy intake (12), yet studies indicate that older adults often exceed this limit (13). Additionally, dietary fibre intake is generally inadequate across all population groups in Europe (14), with older adults particularly benefiting from a daily intake of at least 25 g to alleviate issues such as constipation (15). In NHs, many residents suffer from chronic disease and frailty (16); therefore, it is important that nutritional status is monitored and meals are carefully planned in order to support health and assist in slowing or preventing further decline, which is a burden for individuals and society and also increases care costs.

Gathering data on nutrient intakes is essential to provide valuable insights into the dietary habits of NH residents. NH meals are centrally planned, and residents rely almost entirely on NHs to provide their daily meals. Epidemiological data on NH residents’ nutrition is therefore invaluable for menu planning and aiding policymakers in developing targeted interventions and guidelines to improve the nutrition of older adults in such settings. It is important for NH staff to be familiar with the nutritional challenges of older adults living in NHs and to be trained to intervene effectively, as this is not always the case (17).

The epidemiological data on nutrition in NH in Europe is quite scarce. In Slovenia, there has been no comprehensive epidemiological study investigating the diets of NH residents. In the scope of the NutriCare project, this was addressed nationwide on a representative sample of NH residents. The aim of the present study was to explore the usual dietary intake of energy and macronutrients, as well as the consumption of specific food groups, among NH residents, and compare dietary patterns with the established recommendations. Additionally, the offers in the NH menus were explored.

## Methods

### Study design and selection of participants

This cross-sectional study was conducted as a part of the Slovenian National Research Project NutriCare, which focuses on dietary challenges among nursing home residents. The data was collected in June 2022, September 2022 (10 NHs), December 2022, January 2023, and February 2023 (10 NHs) to include both summer and winter seasons. Ethical approval for the study was obtained from the National Medical Ethics Committee (0120-531/2021/13) on 4.5.2022. The study was registered on clinicaltrials.gov (NCT05389618) (18).

The study was conducted in all 9 health regions, as defined by Slovenian National Institute for Public Health (19). The data on the residential demographic structure of all NH in Slovenia was obtained from the Ministry of Labour, Family, Social Affairs, and Equal Opportunities and used for the selection of NH for this study. Both public NHs and private NHs (with public concession) were included. A total of 20 NHs were included, with at least one from each region. First, the largest NH in each region was invited, while other(s) from the same region were selected using simple randomisation. If the largest regional NH declined participation, an alternative NH was selected using randomisation. The target sample size was set at a minimum of 300 participants, and quota sampling was conducted based on sex and age groups, following the approach used in the EFSA-funded nationally representative dietary survey SI. Menu (20), which included a subsample of community-dwelling older adults (but excluding NH residents). The final sample included *N* = 387 subjects (response rate 54%), representing more than 2% of the population in NHs (in the year 2022 Slovenian NH had altogether 17,939 residents aged 65+ years). For reference, similar national studies in other countries were commonly performed on smaller study samples (21, 22). It should be noted that the study sample was not intended to include bedbound, cognitively, or mentally impaired persons, who also reside in NHs. The study sample was selected from a relatively independent NH subpopulation, requiring low-level care (care category I–IIIa) and being able to give informed consent, which presents more than 80% of the population living in NHs in Slovenia.

After obtaining approval from NH management to conduct the study in the selected NHs, they provided an anonymous list of residents, including data on their care category, age, and sex. Based on predefined quota requirements for age groups (65–80 and over 80 years) and sex (men and women), each NH contributed to the quotas according to their resident composition and the number of residents in the selected care categories. Using simple computer-generated randomisation, and considering the size of the NH and the quota requirements, at least 36 participants, distributed by age and sex, were invited from each NH. Those who accepted the invitation to the study and signed an informed consent form were enrolled. The inclusion criteria were as follows: participants aged 65 years or older, have lived in an NH for at least 3 months before joining the study, and fall into care categories I., II., or III.A (indicating they require little or no personal assistance and are not severely cognitively impaired). Additionally, participants should be able to feed themselves independently or with minimal help and must have signed informed consent. The exclusion criteria were acute illness, being a dialysis patient, having a temporary special diet/ fasting at the time of the study visit, being unable to be weighed by the usual weighing scale or chair weighing scale, not being a full-time resident of an NH, and being in isolation due to COVID-19 measures.

### Variables and data collection

Data was collected from the selected NHs during two study visits, which took place at least 7 days apart. The interviews and anthropometric measurements were performed by the trained researchers. At the first visit, data on age, sex, year of birth, and care category were taken from the NH records and medical records; data on disease and disease history, as well as data on the medications used, were collected. Anthropometric and body composition measurements were also taken. A general questionnaire, adopted and modified from the SI.Menu study (20), was used in the interviews to assess sociodemographic and health status, including eating habits, food allergies, and use of non-prescription drugs and food supplements. Participants were questioned on their physical activity using the International Physical Activity Questionnaire (IPAQ), and the scores were calculated using an established methodology (23). Dietary habits were assessed by two 24-h dietary recall (24HDR) and food frequency questionnaires (FFQ). The first 24HDR was performed on the first visit and on the second visit, the second 24HDR was performed. The participating NHs provided recipes with the ingredients amounts and meal portion amounts for all served meals on each of the 2 days that were questioned in the 24HDR. Additionally, 13 NHs provided these data for 7 days. Data on chronic disease were provided by the NH nurses from participants’ medical records.

### Food consumption data

Data on dietary intake was collected by 24HDR for two non-consecutive days, at least 7 days apart, according to the EFSA guidelines (24). In each NH, the data were collected in one season and both weekdays and weekends were considered, depending on the day of the study visit. For assistance, the NH menus with all dishes and portion sizes for the day of questioning were available, so the interviewers helped the participants recall the dishes on the menu and questioned them on any additional food, beverage, or supplements they consumed on that day. Recording of the consumed amounts of reported foods was supported using portion sizes in the daily menu of particular NH and a picture book (25), which was previously developed and used for the national dietary study SI.Menu. The picture book contains different food products and common dishes, accompanied by six portion sizes. For each reported food, it was also coded whether it was provided by the NH or not (i.e., for foods purchased by individual volunteers, provided by their relatives, etc.). The 24HDR was complemented with the FFQ to collect the data on the frequency of consumption of specific food categories in the past year, enabling us to model usual intakes (26). Altogether, 43 different food categories were included according to the food categorisation system and adopted and modified by Haubrock et al. (27) and the DGE Nutrition Circle (28). For each food category, daily frequencies of consumption were calculated based on the reported consumption frequency (e.g., 3× weekly). For some food categories (milk and dairy products, bread, and cereal products), recommendations for respective food subcategories were aggregated.

The data on dietary intake was analysed using the Open Platform for Clinical Nutrition (29), a web tool, which includes a Slovenian database with nutrient contents of generic and some branded foods (30). The OPEN Platform consists of nutritional composition data for different foods and recipes and allows the input of new recipes. All foods and beverages, reported in the 24HDR, were assigned to the OPEN Platform to obtain nutrient composition data to calculate daily nutrient intakes. The recipes for the dishes on the menu of each of the participating NHs were inputted into the OPEN platform, which assisted in the correct calculation of nutritional composition also for more complex dishes consumed. Nutrients were calculated for foods as prepared for consumption (e.g., cooked pasta).

### Exclusion of mis-reporters

Participants, who did not report both 24HDR (*n* = 7), were not included in the present study because we were not able to calculate their usual dietary intakes. Under- and over-reporting was assessed using the Goldberg method (31). This method assesses the plausibility of participants’ reported energy intake by comparing it to their estimated basal metabolic rate (BMR) and physical activity level (PAL) to evaluate if the reported energy intake aligns with the expected energy expenditure for such a population. The assigned PAL for the population in this study was 1.3, reflecting that the participants mostly exhibited very low physical activity levels, with many being mostly sedentary and some being wheelchair-bound (32). The BMR was estimated according to the Katch–McArdle equation (33) considering lean body mass (LBM). For participants with missing lean body mass data (*n* = 53), we estimated the BMR based on the linear association between values, calculated by two Katch McArdle equations, one considering LBM and the other anthropometric parameters (33) to enable the inclusion of these participants in the analytic sample. Cut-offs, calculated using the Goldberg method, considering a 95% confidence interval were 0.96 and 1.76; 27 under- and 27 over-reporters were excluded.

### Anthropometric and body composition measurements

At the first study visit, height and weight were measured using the Seca 799 medical scale (Seca GmbH, Hamburg, Germany). Waist, hip, arm, and calf circumferences were measured using a soft measuring tape. Body composition was estimated using a Bodystat Multiscan 5,000 multifrequency bioelectrical impedance monitor (Bodystat, Isle of Man, Ireland). This device measures bioelectrical impedance by transmitting mild electrical current (800 μA) across a spectrum of frequencies (5–500 kHz) throughout the subject’s body via four electrodes. Two of the electrodes are positioned on the right hand and two on the right foot. Participants with cardiac pacemakers and those who were unable to get into the position suitable for performing measurements were excluded from this analysis. Bioimpedance techniques have been widely used for estimating body composition due to their non-invasiveness, portability, ease of use, and relatively low operation costs, compared to other techniques (34), such as the MRI and DXA methods (35). Body composition was estimated from the equations used in the Geriatric mode of Bodystat Multiscan 5,000 operation. The estimated lean body mass was further used for the estimation of participants’ BMR. Physical activity was assessed using the short form of the International Physical Activity Questionnaire (IPAQ) (23).

### Data analysis

Usual daily dietary intakes were calculated from the two 24HDR and FFQ. The Multiple Source Method (MSM) was used for modelling variations in the intake distribution between and within days and individuals (27). Such a modelling approach enables the estimation of usual daily intake by correcting variations in food and nutrient intake of an individual by considering the FFQ data, which provide information on long-term dietary habits. FFQ frequencies were obtained by converting the reported frequency of consumption of specific food categories to daily frequencies (e.g., foods consumed twice per week had a daily frequency of 2/7). Participants recorded as true non-consumers reported they never eat foods from specific food categories.

The data on usual intakes of energy, nutrients, and food groups were presented as mean with standard deviation (SD) and median/quartiles or as numbers and percentages per day, for each sex. Usual daily mean (UDM) energy and protein intake were presented in kJ/kg or g/kg body weight, respectively. For statistical analyses, STATA (version 17.0; StataCorp LLC, College Station, TX, USA) and IBM SPSS Version 27 (IBM SPSS, IBM Corp., Armonk, NY) were used.

To obtain the adherence of usual intakes to the recommendations, energy and nutrient intakes were compared to the national reference values, which were adopted from the D-A-CH reference values for energy and nutrient intake (12) and the ESPEN guidelines on clinical nutrition for geriatrics (10). The proportions of the participants with usual intakes below or above the recommended values were calculated. Adherence to the intake of the recommended daily amounts of specific food groups was also presented, according to DGE Nutrition Circle criteria (28). In the case of food categories, for which the recommended amounts referred to weekly consumption (e.g., meat and eggs), these were converted to recommended daily amounts and presented accordingly.

Altogether, 13 NHs also provided daily menus for 7 consecutive days, while 7 NHs provided daily menus for 2 non-consecutive days. However, four daily menus were excluded from the analysis due to their total energy deviating more than two standard deviations from the mean. In total, 108 daily NH menus were included in the analyses of the offered menus in NHs. We modelled the daily macronutrient content data provided by NHs to estimate the macronutrient composition in daily menus and main meals, offered by all NHs. For this purpose, we used linear mixed model analysis with the random effect of NHs on intercepts. Additionally, the generalised linear mixed-effects model analysis was employed to explore differences in main meals to contain at least 25 g of protein among (1) NHs, and (2) main meals (breakfast, lunch, and dinner). We looked at both NHs with random effects on the intercepts set on the type of meal and main meals with random effects of NHs on intercepts.

## Results

Altogether, 378 participants were included in the NutriCare study. For nutrition assessment, seven participants were excluded due to incomplete data on 24HDR and FFQ, and 54 were classified as mis-reporters using the Goldberg method and were excluded from further analysis. The final sample for the present study therefore included 317 participants. The characteristics of the participants are presented in Table 1. The participants’ flowchart is presented in Supplementary Table 3.

**Table 1:** Characteristics of participants in the NutriCare study (Slovenia, 2022–2023).

	Men	Women
*N* (%)	136 (42.9)	181 (57.1)
Care category I; *N* (%)	99 (73)	131 (72)
Care category II; *N* (%)	33 (24)	43 (24)
Care category III; *N* (%)	4 (3)	7 (4)
Age—years; mean (SD)	79.7 (7.8)	83 (7.4)
Height—m; mean (SD)	1.7 (0.09)	1.5 (0.07)
Weight—kg; mean (SD)	82.5 (16.9)	71.8 (15.8)
BMI—kg/m^2^; mean (SD)	29.2	29.7
<18.5 kg/m^2^; *N* (%)	0	3 (2)
18.5–24.9 kg/m^2^; *N* (%)	32 (24)	34 (19)
25–29.9 kg/m^2^; *N* (%)	51 (37)	73 (40)
≥30 kg/m^2^; *N* (%)	53 (39)	71 (39)
BMR (Kcal/day); mean (SD)	1,368 (192)	1,112 (192)
IPAQ		
Low; *N* (%)	51 (38)	65 (36)
Moderate; *N* (%)	74 (54)	110 (61)
High; *N* (%)	11 (8)	6 (3)
MNA; mean (SD)	24.2 (2.9)	24.1 (3.0)
<17; *N* (%)	4 (2)	6 (3)
17–23.5; *N* (%)	58 (35)	68 (31)
24–30; *N* (%)	105 (63)	145 (66)
Number of chronic diseases; mean (SD)	1.7 (1.3)	1.8 (1.4)
Participants with no chronic disease (%)	16.9	18.8
Participants with at least one chronic disease (%)	83.1	81.2

SD, standard deviation; BMI, body mass index; MNA, Mini Nutritional Assessment; BMR, basal metabolic rate; IPAQ, International Physical Activity Questionnaire.

Table 2 presents the descriptive statistics of the usual daily dietary intake of participants. Wide UDM energy intake ranges were observed in both sexes; however, at least half of the population in both sexes had UDM energy intakes below 30 kcal/kg body weight, which is recommended by the ESPEN (10). On the other hand, a high prevalence of high BMI was observed (Table 1).

**Table 2:** Descriptive statistics of usual daily dietary intakes in men and women nursing home residents in the NutriCare study (Slovenia, 2022–2023).

Macronutrients	Men							Women						
	Mean	SD	P5	P25	Median	P75	P95	Mean	SD	P5	P25	Median	P75	P95
Energy (kcal/day)	2,288.7	325.1	1,756.7	2,066.0	2,233.1	2,480.6	2,924.9	2,089.8	277.3	1,642.6	1,921.9	2,077.4	2,272.6	2,524.3
kcal/kg body weight	29.0	6.8	19.0	24.1	28.1	33.8	41.3	29.9	7.8	19.8	24.4	28.8	34.0	44.9
Carbohydrates (g/day)	260.3	45.9	193.6	226.8	257.8	290.1	337.9	247.0	39.1	185.6	216.9	245.6	272.4	311.2
% TEI	45.9	5.4	38.0	42.3	46.0	49.1	54.9	47.3	5.2	38.9	44.0	47.1	50.3	56.7
Total sugars (g/day)	92.7	29.7	58.5	75.1	90.1	104.6	129.9	91.4	21.7	63.0	76.6	89.5	104.0	133.4
% TEI	16.4	4.6	11.2	13.8	15.8	18.2	23.7	17.5	3.9	12.4	14.9	16.8	19.6	24.8
Protein (g/day)	87.9	15.1	62.6	79.7	89.0	97.3	112.4	79.5	14.8	53.7	69.3	80.1	88.3	104.7
g/kg body weight	1.1	0.3	0.7	0.9	1.1	1.3	1.6	1.2	0.3	0.7	0.9	1.1	1.3	1.8
% TEI	15.3	2.0	12.3	14.2	15.2	16.6	18.3	15.3	2.0	12.0	13.9	15.5	16.7	18.6
Total fats (g/day)	92.5	17.7	64.0	80.5	91.1	101.8	123.6	83.0	15.5	59.5	72.2	81.2	93.0	110.4
% TEI	36.0	5.2	29.1	32.3	35.3	39.6	45.0	35.8	5.0	27.9	32.1	35.9	39.5	43.9
Saturated fats (g/day)	34.3	10.1	18.9	29.2	33.0	37.6	49.9	30.4	8.3	19.3	25.0	29.5	33.9	48.0
% TEI	13.5	3.6	9.1	11.3	13.0	15.2	18.4	13.1	3.2	8.9	10.7	12.8	14.9	19.6
% Total fats	36.4	4.8	29.4	33.6	36.7	39.6	43.3	36.7	5.4	28.2	32.8	37.2	40.5	44.4
Dietary fibres (g/day)	20.4	4.4	13.2	17.0	19.9	23.4	28.8	19.5	4.1	13.3	16.9	19.0	22.4	26.6
Total water (ml/day)	2,058.8	412.6	1,458.5	1,796.3	2,016.2	2,301.0	2,819.7	1,980.3	344.6	1,522.8	1,728.2	1,951.5	2,194.2	2,620.1

Total sugars: all types of sugars present in foods (added and naturally occurring). Total water: total water intake from beverages and solid foods. SD, standard deviation; P, percentile; TEI, total energy intakes.

Daily carbohydrate intake below the recommended 50% of daily energy intake was observed in 81% of men and 66% of women (Table 3). Low mean dietary fibre intake was observed, which was approximately 20 g daily for both sexes (Table 2). Less than 20% of the participants had dietary fibre intakes of at least 25 g daily (Table 3). Approximately 90% of the participants had fat intakes above 30% of total daily energy intake (Table 3).

**Table 3:** Proportions of men and women nursing home residents meeting reference values for specific food categories, energy, and macronutrients in the NutriCare study (Slovenia, 2022–2023).

	Men			Women			Dietary reference values (DRV)
	% below min DRV	In the DRV range	% above max DRV	% below min DRV	In the DRV range	% above max DRV	
Food categories							
Milk and dairy products	20.59	13.24	66.18	21.55	10.50	67.96	250–310 g/day
Vegetables	92.7		7.4	96.7		3.3	>250 g/day
Fruits	93.4		6.6	85.6		14.6	>250 g/day
Potatoes, pasta, rice	95.6	4.4		98.9	1.1		200–250 g/day
Bread and cereal products	94.9	5.1		99.5	0.5		200–310 g/day
Fish and fish products	92.6		7.4	87.9	1.1	11	22–32 g/day
Meat (fresh and processed)			100	0.5	1.7	97.8	43–86 g/day
Eggs	41.9		58.1	54.1		45.9	<26 g/day
Oils and fats	33.1	64	2.9	54.1	45.9		25–45 g/day
Water and non-alcoholic beverages	97.1	2.9		98.3	1.7		>1,500 mL/day
Macronutrients							
Energy intake	61.2		38.8	53.4		46.6	> 30 kcal/kg BM
Carbohydrates	80.6		19.4	66.3		33.7	>50% of total energy intake
Proteins (criteria 1)	40.3		59.7	35.4		64.6	>1 g/kg BM
Proteins (criteria 2)	70.5		29.5	61.8		38.2	>1.2 g/kg BM
Total fats	5.8		94.2	10.1		89.9	< 30% of total energy intake
Dietary fibre (criteria 1)	82.7		17.3	91.6		8.4	>25 g/day
Dietary fibre (criteria 2)	97.1		2.9	99.4		0.6	>30 g/day
Total water	86.3		13.8	59.7		40.3	>2,500/2,000 mL/day

Recommended daily intakes taken from the DGE nutrition circle (28), converted to daily recommended amounts of specific food categories; recommended total water intake adopted from EFSA dietary reference values for water (men: 2,500 mL/day, women: 2,000 mL/day) (58).

The UDM protein intake was 1.1 g/kg body weight for men and 1.2 g/kg body weight for women (Table 2). However, as presented in Table 4, 40% of men and 35% of women do not meet the minimum daily reference protein intake of 1 g/kg body weight. Given the high prevalence of chronic diseases among the NH residents, the recommended daily protein intake according to ESPEN (10) would be closer to 1.2 g/kg body weight. The median intake of 1.1 g/kg body weight for both sexes suggests that more than 50% of participants have suboptimal daily protein intakes (Table 2). In fact, 70% of men and 62% of women had usual daily protein intakes below this threshold (Table 3). A high range of UDM protein intakes was noticed in both sexes, from 0.5 g/kg body weight to more than 2 g/kg body weight, indicating a high variability of UDM protein intakes per kg body weight among participants (Table 2).

**Table 4:** Linear mixed model mean (CI) estimates of macronutrient composition of daily meals served in nursing homes in the NutriCare study (Slovenia, 2022–2023).

Macronutrients	All meals	Breakfast	Morning snack	Lunch	Afternoon snack	Dinner
Energy (kcal/day)	2,013 ± 114	621 ± 27^a^	120 ± 46	743 ± 27^b^	172 ± 32	479 ± 27^c^
Carbohydrates (g/day)	231.8 ± 16.4	79.3 ± 4.2^a^	24.0 ± 8.5	70.6 ± 4.2^b^	25.63 ± 3.4	54.9 ± 4.3^c^
Total fats (g/day)	82.3 ± 5.6	23.5 ± 1.9^a^	1.6 ± 2.1	34.3 ± 2.0^b^	5.6 ± 2.2	20.3 ± 2.0^c^
Protein (g/day)	80.0 ± 5.0	22.3 ± 1.7^a^	2.0 ± 1.2	35.3 ± 1.7^b^	4.5 ± 1.6	18.4 ± 1.7^c^
Protein-animal source (g/day)	49.6 ± 3.5	11.9 ± 1.7^a^	0.7 ± 0.9	24.1 ± 1.6^b^	2.9 ± 0.9	11.5 ± 1.6^a^
Protein-plant source (g/day)	29.8 ± 2.3	10.4 ± 0.7^a^	1.11 ± 1.0	10.9 ± 0.7^a^	1.59 ± 0.9	6.85 ± 0.7^b^
Dietary fibre (g/day)	18.7 ± 2.2	3.8 ± 0.6^a^	2.0 ± 0.9	8.7 ± 0.7^b^	2.1 ± 0.5	4.2 ± 0.7^a^

Morning and afternoon snacks were not included in the pair comparison analysis. Superscript letters denote significant differences (*p* < 0.05) in the marginal means between the main meals.

Looking into the food categories and their amounts consumed among the NH residents, Table 3 and Supplementary Table 1 show that according to the dietary reference values, particularly meat was consumed in excess amounts; 100% of men and 98% of women exceeded the maximum reference value of 86 g meat daily, with a mean daily amount of 137 and 114 g, respectively. On the other hand, at least 85% of women and more than 90% of men did not meet the recommended daily intakes for fruit, vegetables, potatoes, pasta, rice, bread, cereal products, and fish.

The results from the linear mixed effects models determining the available macronutrient composition in daily menus and the mean comparison analysis of the meal composition using the random effect of NHs on intercepts are presented in Table 4. The mean modelled energy content of daily NH menus was 2.013 ± 114 kCal. Approximately one-third of energy intake was from breakfast, a third from lunch, a quarter from dinner, and the rest from snacks. The mean amount of protein per meal was the highest for lunch (35.5 ± 1.7 g) and the lowest for dinner (18.4 ± 1.7 g). The dominating source of protein was animal protein, which was most noted in lunch and was significantly higher than in breakfast and dinner. Breakfast and lunch had similar content of plant-sourced proteins, while at dinner, it was significantly lower. The mean daily amount of meat and meat products offered in NH menus was 167 g, which was well above the recommendations (Supplementary Table 2). On the other hand, the total mean daily amounts of fruits and vegetables on NH menus were below 400 g. The foods, consumed from outside sources (not offered through NH menus) presented approximately 1% of daily energy consumption. These were mostly coffee, soft drinks, sweets, and fruit.

## Discussion

Although the analysis of UDM energy intakes among NH residents might suggest a generally satisfactory state, a closer examination revealed considerable variability within the population, which was also evident in UDM macronutrient intakes. A notable concern arises from the fact that the mean BMI of participants exceeded 29 kg/m^2^ in both sexes, with approximately 40% of participants having a BMI > 30 kg/m^2^, indicating obesity according to the World Health Organisation (WHO) criteria (36). Although meta-analyses did not show a significant link between increased mortality and overweight in older adults, going beyond a BMI of 28 kg/m^2^ put a considerable proportion of participants at risk (37). The high prevalence of obesity highlights an important health issue, supposedly originating mainly in imbalances in dietary intake and physical activity. Due to the predominantly sedentary lifestyles of NH residents, the introduction of suitable physical activity is crucial for maintaining their functionality and independence and preventing various diseases, particularly sarcopenic obesity (38). In this population, low BMI and malnutrition are serious concerns, but our study did not find them to be significant issues based on the MNA results (only 3% of malnourished participants), probably mainly due to the characteristics of our study sample (39), which included residents in need of low-level care.

The distribution proportions of UDM macronutrient intakes indicate an opportunity to better align with the established recommendations for macronutrient intake. In older adults, adequate protein intake is crucial for maintaining muscle mass, which tends to decline during this stage of life (40). As shown in Table 3, daily protein intakes were not optimal for many participants. For example, 40% of men and 35% of women failed to meet even the lowest threshold for daily protein intake (1 g/kg BM), compared to 40% of community-dwelling Slovenian older men and 21% of women (20). This could also be related to the variations in body weight of the participants. As dietary protein intakes should be individually adjusted, considering health and nutritional status, the “one-fits-all” approach could hardly suit all NH residents’ protein requirements. This is particularly pronounced in those with higher body weight. In such cases, the recommended protein daily intake (e.g., >1.2 g/kg BM) could be difficult to achieve in practice, especially through communal meals alone, as the protein quantities required could be very high. On the other hand, such high protein quantities could pose a risk for some residents, for example, those with kidney failure. This highlights the importance of monitoring the nutritional status of NH residents and employing individual approaches.

A substantial proportion of the total energy intake (TEI) was shown to be derived from fats, indicating that daily menus, offered in NH exhibit an excessive fat content (37% of energy intake). Less than 10% of NH residents had appropriate daily fat intakes up to 30% of daily energy intake, with men being more exposed. This can be linked to the high consumption of meat and dairy products among NH residents. High dietary fat intakes and imbalances in the type of consumed fats should not be overlooked, as this can potentially influence various health outcomes in older adults, including cognitive function (41), metabolic syndrome (42), and risk of fractures (43). Regarding the fact that approximately 13% of TEI was from saturated fats, balancing meals by including more unsaturated fats would be beneficial. Considering the high prevalence of individuals with high BMI in our sample, more attention should be paid to the fat content of the meals, particularly the type of fats contained in the meals.

Suboptimal carbohydrate and fibre intakes were noted. Inadequate intake of less-refined carbohydrates is associated with low dietary fibre intake, observed in more than 80% of men and 90% of women who consume dietary fibre below the daily threshold of 25 g (10). Generally, the UDM fibre intakes in our study were even lower than in Slovenian community-dwelling older adults (44), where 77% of men and 65% of women had UDM fibre intakes below 25 g. It should be noted that 25 g of dietary fibre daily is the lowest threshold for normal laxation (45), while the optimal daily intake would be 30 g. Regarding the fact that older adults, particularly institutionalised, often have constipation issues (46), it would be crucial to adjust dietary fibre intakes to support normal laxation. For example, it has been shown that dietary fibre intervention in NH residents led to the discontinuation of laxative use in more than half of the users and significantly improved their overall wellbeing (46). Adding more fruits and vegetables to the diet could support this, as currently, approximately 90% of participants do not meet the daily recommendations for these food groups, which is set at >250 g/day (28). Moreover, a higher intake of wholegrain bread and cereal products would be a positive addition to their daily diet and fibre intake.

Adequate hydration was found to be a problem, especially among men. In older adults, this is often a concern, as many of them do not feel thirsty, have incontinence issues, or simply forget to drink. NH residents should regularly be offered drinks and encouraged to consume fluids by NH staff (47).

UDM macronutrient intakes of the participants reflected what we found in the analysis of NH menus. In general, the menus were not optimally balanced (Table 4). As NH residents consume little food from outside sources, NH meals should well suit their dietary needs. Our findings showed that the NH menus failed to provide a balanced macronutrient intake to meet the needs of the NH residents optimally, even though Slovenia has implemented recommendations for meal planning in NHs already in the year 2020 (48). The main issue observed was that the NH daily menus were excessive in fat (37% energy—estimated) and rather scarce in quality carbohydrates (46% energy—estimated), affecting the adequate daily intake of fibre. If, for various reasons, daily protein intakes in NH residents cannot be individually adjusted, meals should at least contain the recommended amount of protein (25–30 g protein per meal) (11, 48). Breakfast and dinner had a particularly low protein content, with a mean below 25 g. We observed significant differences in the likelihood of reaching a minimum of 25 g protein per meal between main meals (*F* = 37.7; df = 2; *p* < 0.001) as well as between NHs (*F* = 1.8; df = 19; *p* = 0.017).

The observed macronutrient intakes essentially mirrored the consumption patterns of specific food groups. The general impression was that NH menus should try to focus on introducing more foods of plant origin. The menus were heavy on foods of animal origin, especially dairy products and meat, but lacked vegetables, fruits, and cereals to balance the nutrient intakes. Although meat is an important source of good quality protein and micronutrients, replacing some of the animal protein and introducing more foods of plant origin, including legumes and nuts, could not only add to the protein content of meals but also add more less-refined carbohydrate sources and dietary fibre to the existing meals, contributing to more balanced diets (49). Foods of animal origin can also be high in fat, which is unfavourable for a healthy diet. Higher dietary fibre intake could be achieved by replacing refined grains, such as white bread and pasta, with whole grains. Meals could include more vegetable side dishes and salads, as well as fruits. Furthermore, reducing the use of foods high in saturated fat by offering leaner cuts of meat, limiting high-fat dairy, and using plant-based oils such as olive oil for cooking would help lower the overall fat content and its quality, which is particularly important given the BMIs observed in this sample. By introducing such changes, NH staff could create more balanced menus that better address the observed discrepancies and promote healthier dietary patterns.

Similar issues in macronutrient and food group composition of dietary intakes of NH residents have been also highlighted in previous studies (50, 51). The main issues of concern were usually low energy and protein intake (22, 52, 53) and also high fat and low dietary fibre intakes (50, 54, 55). However, when comparing and interpreting results from different studies, it is important to be cautious due to the differences in the methodology of data collection and analysis. Although similar methods are used for the assessment of dietary intakes—usually 24HDR or food diaries, many studies report habitual (short term) dietary intakes, while in our study, usual dietary intakes were reported, which reflect long-term dietary habits. Regardless of methodological differences, the observed trends in nutrient intakes often point in the same direction, as mentioned above. As NH residents almost completely rely on NH provision of food, more attention should be paid to include more beneficial food groups currently lacking in their diet.

The importance of including qualified professionals, such as dietitians and clinical nutrition experts, in the multidisciplinary NH teams has been continuously highlighted (56, 57). Only a comprehensive approach and individual monitoring could efficiently combat malnutrition and related preventable health problems in the NH population. Unfortunately, nutrition professionals are currently rarely actively included in the care of Slovenian NH residents. To deliver high-quality care in NHs and avoid additional costs associated with poor nutrition among residents, addressing this issue should be a top priority. In any case, the nutritional status of NH residents should be monitored using established tools, such as the MNA and the Global Leadership Initiative on Malnutrition (GLIM) (58) criteria to identify emerging nutrition-related issues to act promptly and appropriately.

The present study was the first nationally representative study focussed on investigating the nutrition of NH residents in Slovenia. It is one of the few studies comprehensively addressing the nutrition of the older adults in long-term residential institutions with national representativeness. The cross-sectional study design allowed for a snapshot of dietary patterns among this vulnerable population focussing on usual macronutrient intakes and the consumption of various food groups. Most of the studies on the nutrition of NH residents used only the MNA for the assessment of nutritional status, without using tools that allow the estimation of usual dietary intakes. The present study included relatively independent NH residents requiring low-level care, who well-presented most of the Slovenian NH population and were not bedbound. Such a population is characteristically closer to community-dwelling older adults or those living in residential homes; however, the latter is not very common in Slovenia. NH population with low-level care requirements allows for the implementation of improvement strategies to practice, maintain health, and prevent or delay advancing in care assistance. The latter is linked to health decline and the loss of independence, which causes significantly higher costs of care and staff requirements (59). The findings present valuable insights that could assist in further meal planning and areas for potential improvement with the aim of providing better care for older adults. A limitation that should be considered is that not all the NHs provided daily menus for the whole week; therefore, for mean macronutrient meal composition analysis, weights were used. Another limitation is that physical activity was assessed using self-reported IPAQ scores, which should be interpreted with caution. For a more specific assessment of the physical activity, objective methods should be employed such as accelerometers or pedometers. These devices would provide more reliable data, minimising the subjective inaccuracies that accompany self-reporting.

Future studies should explore challenges in achieving the proper nutritional status of distinct subgroups of NH residents, such as those with specific diseases or feeding-related difficulties with the aim to optimise their nutrition according to the specific requirements. The variations in NH menus among NHs across the country could also be assessed. Additionally, interventions that could improve nutrient status, health, and quality of life should be explored. Furthermore, given that NHs facilitate communal meals, there is a need to investigate optimal strategies for accommodating the diverse dietary requirements of residents within a communal dining framework.

## Conclusion

The results of the present study showed that the diet of Slovenian NH residents could be improved. Notable variability in macronutrient intakes was observed; men were found particularly at risk for an imbalanced diet. The prevalence of high BMI reflects the discrepancies between energy intake and expenditure. The diets of NH residents were high in fats and foods of animal origin (meat and dairy) and low in less-refined carbohydrates and dietary fibre, due to insufficient intake of fruits, vegetables, and cereals. The protein content of meals could be enhanced, especially for breakfast and dinner. NH residents consume little food from sources outside the NH, and their macronutrient and food group intakes well reflect the composition of menus served in NHs. Careful menu planning and monitoring of the nutritional status of NH residents should be implemented to avoid health complications and higher care costs. Qualified staff capable of implementing individualised, tailored nutritional monitoring and interventions is needed to ensure optimal care for NH.

## Data Availability

The raw data supporting the conclusions of this article will be made available by the authors, without undue reservation.
