# Supplementary material for: Nutrition among nursing home residents: results from the NutriCare study
Source: Front Nutr. 2024 Nov 11;11:1423658. doi: 10.3389/fnut.2024.1423658 (PMC11586171; doi:10.3389/fnut.2024.1423658)

Supplementary Material

**
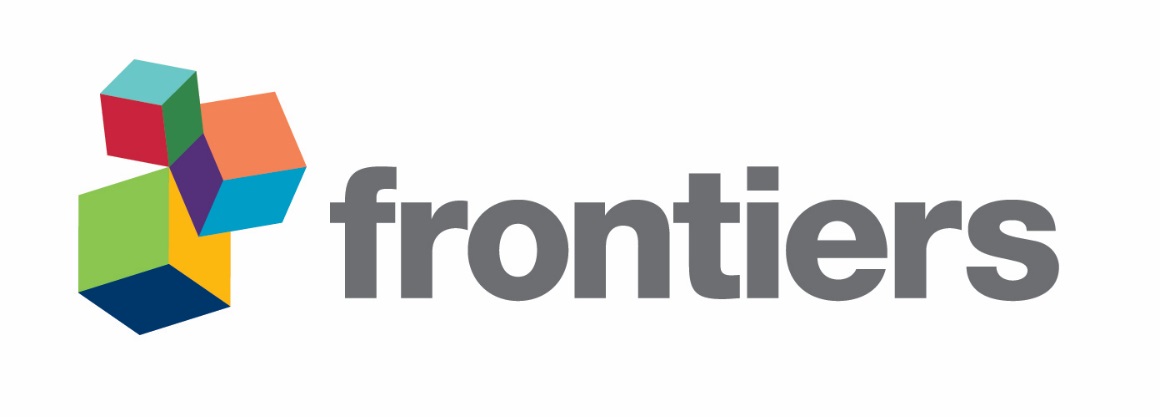
**

**Supplementary table 1:** Descriptive statistics of usual intakes of specific food categories in male and female nursing home residents in the NutriCare study (Slovenia, 2022-2023)

| **Food groups (g or ml/day)** | **Male** | | | | | | | **Female** | | | | | | |
| --- | --- | --- | --- | --- | --- | --- | --- | --- | --- | --- | --- | --- | --- | --- |
|  | Mean | SD | P5 | P25 | Median | P75 | P95 | Mean | SD | P5 | P25 | Median | P75 | P95 |
| **Milk and dairy products** | 357.0 | 128.6 | 159.3 | 265.3 | 352.4 | 445.4 | 578.6 | 356.0 | 118.6 | 159.1 | 269.0 | 354.9 | 427.1 | 562.0 |
| Milk | 290.8 | 117.3 | 72.4 | 223.5 | 302.8 | 366.3 | 468.3 | 290.8 | 111.0 | 81.8 | 222.6 | 300.3 | 363.9 | 459.0 |
| Fermented milk products | 73.8 | 74.7 | 0.0 | 0.0 | 50.4 | 149.2 | 195.1 | 74.3 | 78.1 | 0.0 | 0.0 | 46.1 | 155.5 | 176.8 |
| Cheese | 31.2 | 27.3 | 0.0 | 0.0 | 37.9 | 53.3 | 73.2 | 26.8 | 22.8 | 0.0 | 0.0 | 35.1 | 44.1 | 58.9 |
| **Vegetables** | 158.3 | 64.9 | 61.6 | 118.0 | 144.0 | 204.2 | 288.0 | 142.0 | 53.3 | 68.3 | 103.5 | 137.5 | 168.4 | 240.6 |
| **Fruit** | 154.8 | 75.7 | 0.0 | 126.2 | 168.6 | 198.6 | 259.9 | 184.3 | 73.2 | 0.0 | 159.4 | 192.3 | 223.0 | 300.2 |
| **Legumes** | 23.25 | 31.11 | 0.00 | 0.00 | 12.91 | 29.44 | 90.49 | 16.87 | 26.50 | 0.00 | 0.00 | 0.00 | 23.80 | 70.15 |
| **Nuts and seeds** | 2.3 | 6.0 | 0.0 | 0.0 | 0.0 | 0.0 | 17.3 | 1.6 | 4.5 | 0.0 | 0.0 | 0.0 | 0.0 | 12.2 |
| **Potatoes** | 97.1 | 45.7 | 0.0 | 98.3 | 111.9 | 124.9 | 137.7 | 86.0 | 38.9 | 0.0 | 85.5 | 97.4 | 108.1 | 124.2 |
| **Bread** | 116.5 | 27.4 | 79.9 | 97.4 | 112.1 | 139.8 | 162.7 | 93.5 | 20.7 | 69.5 | 75.9 | 91.6 | 105.9 | 132.5 |
| Bread (brown, wholegrain) | 74.5 | 52.5 | 0.0 | 0.0 | 73.9 | 117.9 | 155.9 | 61.3 | 42.9 | 0.0 | 0.0 | 69.5 | 88.7 | 116.4 |
| Bread (white) | 66.4 | 49.1 | 0.0 | 27.3 | 70.6 | 102.5 | 149.0 | 55.1 | 41.9 | 0.0 | 0.0 | 65.8 | 84.9 | 119.4 |
| **Cereal and cereal products** | 61.7 | 16.4 | 31.7 | 50.1 | 63.3 | 72.1 | 90.0 | 54.9 | 14.8 | 33.2 | 45.7 | 53.6 | 65.7 | 79.1 |
| Unprocessed cereals | 38.9 | 6.1 | 32.3 | 37.0 | 39.1 | 42.5 | 45.6 | 35.9 | 6.4 | 30.5 | 34.1 | 36.4 | 38.8 | 42.2 |
| Pasta | 28.8 | 20.1 | 0.0 | 18.5 | 25.9 | 37.1 | 62.3 | 23.2 | 19.7 | 0.0 | 0.0 | 21.4 | 36.7 | 59.2 |
| Rice | 15.3 | 21.4 | 0.0 | 0.0 | 0.0 | 35.8 | 56.7 | 14.9 | 20.8 | 0.0 | 0.0 | 0.0 | 27.5 | 54.7 |
| **Meat and meat products** | 137.1 | 13.0 | 115.1 | 127.5 | 138.0 | 144.3 | 159.6 | 114.3 | 16.5 | 93.2 | 104.5 | 114.2 | 124.7 | 140.6 |
| Red meat | 74.7 | 40.8 | 0.0 | 68.5 | 90.5 | 100.6 | 116.8 | 62.9 | 35.5 | 0.0 | 60.4 | 75.6 | 86.0 | 98.8 |
| Poultry | 73.0 | 55.4 | 0.0 | 0.0 | 99.3 | 115.7 | 144.2 | 51.8 | 45.8 | 0.0 | 0.0 | 69.3 | 93.8 | 111.8 |
| Processed meat | 48.5 | 42.7 | 0.0 | 0.0 | 46.5 | 77.1 | 117.1 | 39.0 | 38.8 | 0.0 | 0.0 | 38.4 | 66.2 | 115.1 |
| **Fish and fish products** | 6.3 | 20.6 | 0.0 | 0.0 | 0.0 | 0.0 | 79.6 | 7.5 | 20.0 | 0.0 | 0.0 | 0.0 | 0.0 | 60.8 |
| **Eggs** | 33.4 | 22.7 | 0.0 | 17.5 | 28.6 | 51.0 | 78.8 | 26.4 | 18.8 | 0.0 | 17.5 | 24.0 | 36.7 | 57.9 |
| **Fats and oils** | 29.4 | 8.4 | 16.0 | 22.8 | 29.2 | 35.8 | 43.6 | 25.0 | 7.3 | 14.2 | 19.5 | 24.3 | 30.6 | 38.0 |
| Vegetable fats | 18.9 | 6.7 | 7.1 | 14.0 | 19.2 | 23.4 | 29.3 | 15.8 | 5.9 | 6.8 | 10.8 | 15.6 | 20.1 | 25.9 |
| Animal fats | 14.8 | 8.0 | 0.0 | 16.5 | 18.7 | 19.4 | 21.2 | 12.3 | 8.0 | 0.0 | 0.0 | 16.7 | 17.9 | 19.5 |
| **Water and non-alcoholic beverages** | 826.2 | 288.7 | 422.8 | 636.4 | 775.6 | 1012.7 | 1393.9 | 876.7 | 278.9 | 474.3 | 665.8 | 869.3 | 1079.9 | 1321.3 |
| Water | 772.3 | 272.3 | 390.7 | 577.4 | 721.2 | 950.2 | 1235.3 | 827.3 | 264.9 | 414.1 | 642.3 | 799.2 | 1020.5 | 1256.4 |
| **Coffee and tea** | 105.3 | 74.5 | 0.0 | 61.2 | 86.4 | 130.6 | 249.4 | 97.8 | 62.4 | 46.4 | 57.1 | 78.7 | 118.9 | 232.5 |
| **Alcoholic beverages** | 109.6 | 198.0 | 0.0 | 0.0 | 0.0 | 125.9 | 540.1 | 21.8 | 71.2 | 0.0 | 0.0 | 0.0 | 19.0 | 111.3 |
| **Sweets** | 48.8 | 15.9 | 24.6 | 39.4 | 47.8 | 57.2 | 75.8 | 49.6 | 14.5 | 30.7 | 41.8 | 48.1 | 56.7 | 73.6 |
| **Savoury snacks** | 0.6 | 3.9 | 0.0 | 0.0 | 0.0 | 0.0 | 0.0 | 1.9 | 13.0 | 0.0 | 0.0 | 0.0 | 0.0 | 10.4 |
| **Sauces** | 0.9 | 3.8 | 0.0 | 0.0 | 0.0 | 0.0 | 6.5 | 0.9 | 3.5 | 0.0 | 0.0 | 0.0 | 0.0 | 6.5 |
| **Spices and condiments** | 14.8 | 21.6 | 2.2 | 5.1 | 10.8 | 15.6 | 33.7 | 11.8 | 16.6 | 3.0 | 4.9 | 8.7 | 12.0 | 22.5 |

P – percentile; SD – standard deviation

**Supplementary table 2**: Descriptive statistics of specific food categories offered in nursing home daily menus (N=108)

| **Food categories (g or ml/day)** | **Mean** | **SD** | **Median** | **P 25** | **P 75** | **P 95** |
| --- | --- | --- | --- | --- | --- | --- |
| Coffee and tea | 30.7 | 66.8 | 4.7 | 2 | 20 | 208 |
| Bread (all types) | 165.2 | 62.7 | 138.6 | 120 | 200 | 300 |
| Meat and meat products (red meat, processed meat and poultry) | 167.0 | 78.4 | 170 | 110 | 210 | 330 |
| Milk and milk products (milk, fermented milk, cheese, etc.) | 408.1 | 180.9 | 400 | 265 | 500 | 754.7 |
| Vegetable fats | 23.6 | 11.7 | 22 | 15 | 32.7 | 45.0 |
| Fruits (all types) | 176.1 | 86.6 | 160 | 130 | 198.1 | 380 |
| Sweets and sweet products | 44.3 | 39.9 | 35 | 21 | 50.6 | 104 |
| Soft drinks and non-alcoholic beverages | 119.0 | 115.2 | 152 | 5 | 200 | 305 |
| Drinking water | 340.4 | 717.6 | 250 | 180 | 379 | 570 |
| Vegetables (all types) | 186.7 | 99.0 | 172.3 | 109 | 251.5 | 360 |
| Cereal and cereal products | 85.0 | 54.0 | 77 | 45 | 109 | 180 |
| Animal fats | 21.1 | 11.3 | 20 | 15 | 27 | 40 |

P – percentile; SD – standard deviation

**Supplementary figure 1: Participant flow chart**


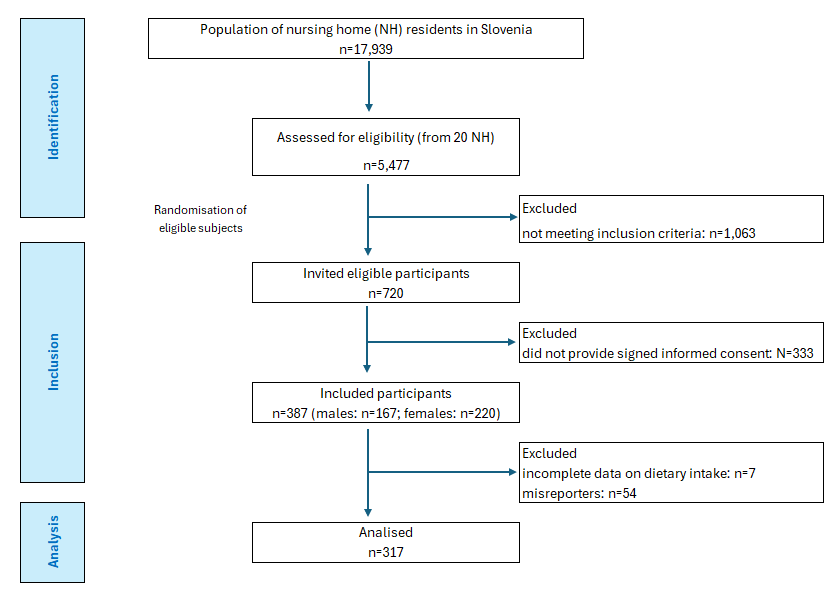

Supplement: Supplementary file 1 [file Data_Sheet_1.docx]
